# Serious mental illnesses associated with receipt of surgery in retrospective analysis of patients in the Veterans Health Administration

**DOI:** 10.1186/s12893-015-0064-7

**Published:** 2015-06-18

**Authors:** Laurel A. Copeland, John E. Zeber, Edward Y. Sako, Eric M. Mortensen, Mary Jo Pugh, Chen-Pin Wang, Marcos I. Restrepo, Julianne Flynn, Andrea A. MacCarthy, Valerie A. Lawrence

**Affiliations:** Veterans Affairs: Central Texas Veterans Health Care System, Center for Applied Health Research, 2102 Birdcreek Drive, Temple, TX 76502 USA; Baylor Scott & White Health: Center for Applied Health Research, 2102 Birdcreek Drive, Temple, TX 76502 USA; Texas A&M Health Science Center, College of Medicine, Bryan, TX USA; UT Health Science Center San Antonio, San Antonio, TX USA; Veterans Affairs: North Texas Veterans Health Care System, 4500 S. Lancaster Rd, Dallas, TX 75216 USA; UT Southwestern Medical Center, Dallas, TX USA; Veterans Affairs: South Texas Veterans Health Care System, 7400 Merton Minter (11c6), San Antonio, TX 78229 USA

**Keywords:** Bipolar disorder, Depressive disorder, Post-traumatic stress disorder, Schizophrenia, Surgical procedures, Operative, Veterans

## Abstract

**Background:**

The STOPP study (Surgical Treatment Outcomes for Patients with Psychiatric Disorders) analyzed variation in rates and types of major surgery by serious mental illness status among patients treated in the Veterans Health Administration (VA). VA patients are veterans of United States military service who qualify for federal care by reason of disability, special service experiences, or poverty.

**Methods:**

STOPP conducted a secondary data analysis of medical record extracts for seven million VA patients treated Oct 2005-Sep 2009. The retrospective study aggregated inpatient surgery events, comorbid diagnoses, demographics, and postoperative 30-day mortality.

**Results:**

Serious mental illness -- schizophrenia, bipolar disorder, posttraumatic stress disorder, or major depressive disorder, was identified in 12 % of VA patients. Over the 4-year study period, 321,131 patients (4.5 %) underwent surgery with same-day preoperative or immediate post-operative admission including14 % with serious mental illness. Surgery patients were older (64 vs. 61 years) and more commonly African-American, unmarried, impoverished, highly disabled (24 % vs 12 % were Priority 1), obese, with psychotic disorder (4.3 % vs 2.9 %). Among surgery patients, 3.7 % died within 30 days postop. After covariate adjustment, patients with pre-existing serious mental illness were relatively less likely to receive surgery (adjusted odds ratios 0.4-0.7).

**Conclusions:**

VA patients undergoing major surgery appeared, in models controlling for comorbidity and demographics, to disproportionately exclude those with serious mental illness. While VA preferentially treats the most economically and medically disadvantaged veterans, the surgery subpopulation may be especially ill, potentially warranting increased postoperative surveillance.

## Background

For veterans without monetary or insurance resources, the choice of hospital in which to undergo inpatient (non-ambulatory) surgical treatment may be outside their control. In the Veterans Health Administration (VA), the largest integrated healthcare system in the United States, patients become eligible for VA care as veterans of US military service by reason of medical or psychiatric disabilities, low income, or factors related to their military service such as receiving a Purple Heart. In the face of limited resources, the VA prioritizes offering care to the most disadvantaged veterans including those with a serious mental illness, recognizing the lack of options that comes with an inability to work, to maintain social ties, or to care for oneself [1]. The quality of their inpatient care then becomes a matter of national interest, not only to provide optimal care for those who have served their country but also to ensure good fiscal management of the taxpayer dollar which supports the VA system.

While the Veterans Administration Surgical Quality Improvement Program (VASQIP) was established to collect meticulously detailed perioperative data and to monitor the quality of surgical outcomes within the Veterans Health Administration, neither VASQIP nor its counterpart in the American College of Surgeons collects preoperative psychiatric diagnoses [2, 3]. As a result, the potential effect of psychiatric illness on perioperative care is largely unevaluated.

That pre-existing psychiatric disorders may play a role in surgical intervention or outcomes is suggested by the large literature on high rates of physical comorbidity for persons with serious mental illness. The Veterans Health Administration defines serious mental illness as schizophrenia, bipolar disorder, post-traumatic stress disorder (PTSD), or major depressive disorder (MDD), and allocates resources specifically for treating patients with serious mental illness [4]. However, a systematic review of evidence published from 1966 to August 2007 regarding postoperative clinical outcomes for patients with serious mental illness identified only 12 studies – 10 of patients with schizophrenia and two concerning patients with major depressive disorder [5]. Three reports suggested that patients with schizophrenia have more postoperative complications including death and may present at later stage of surgical disease [6–8]. Two found that pre-operative discontinuation of psychotropic medication in patients with schizophrenia or depression resulted in more postoperative delirium, confusion, and psychiatric symptoms than when medications were continued [9, 10]. The systematic review found no studies of clinical outcomes of surgery for patients with preoperative PTSD or bipolar disorder. Subsequent papers have noted (a) 25 % of patients admitted to VA intensive care units (ICU) had preoperative mental illness, (b) adjusted 30-day mortality rates were higher for ICU patients with preoperative depression or anxiety, and (c) patients with schizophrenia had elevated rates of postoperative complications including death [11, 12]. The frequency with which patients with serious psychiatric conditions undergo surgery also appears to be undocumented. Surgical intervention represents a crucial dimension to understanding the medical comorbidity and medical care needs of seriously mentally ill patients.

There are important reasons to suspect that patients with serious mental illness may fare poorly perioperatively. First, because of sedentary lifestyle, diet, high smoking rates, suicidality, and genetic susceptibility, persons with serious mental illness age early and are at the leading edge of their age cohort in terms of multi-morbidity and mortality. They are essentially a decade “older” physiologically than their counterparts [13–15]. Second, because of poor insight, difficulty articulating symptoms, and for those with schizophrenia higher pain thresholds, they may present at a later stage of surgical disease, increasing their risk of postoperative complications [16, 17]. Third, because of poor treatment engagement and care coordination challenges, they may receive less preventive care and less management of comorbid medical conditions such as diabetes and coronary artery disease, resulting in surgical conditions that might have been prevented (e.g., lower extremity amputation due to late manifestations of peripheral arterial disease rather than bypass or stent procedures at earlier stages of disease) [14]. As a corollary, they may present to any operation with higher perioperative risk due to the higher burden of comorbid illness.

This retrospective cohort study offers the first comprehensive look at the types and relative rates of surgery experienced by VA patients with and without the four serious mental illnesses defined above. The purpose of this study was to describe (a) the population of patients undergoing inpatient surgery in the VA and variation in surgery patients’ characteristics by mental illness status, (b) types and rates of surgeries by mental illness status, and (c) post-operative mortality and complications by serious mental illness status.

## Methods

### Sample and data sources

The Surgical Treatment Outcomes for Patients with Psychiatric Disorders (STOPP) study used administrative data extracts from the VA’s all-electronic medical record system for the fiscal years (FY) 2006-2009. The study was approved by the institutional review boards at South Texas Veterans Health Care System (#10-187H) and Central Texas Veterans Health Care System (#00412) prior to initiation; waivers of informed consent and HIPAA authorization were granted. Data were aggregated to examine serious mental illness diagnosis and receipt of qualifying inpatient operations. Veterans using the VA during that time frame were eligible for inclusion in the study (*n* = 7.1 million persons), including the subset who experienced inpatient surgical treatment (*n* = 321,131). Qualifying inpatient operations were invasive procedures requiring either preoperative or immediate (same-day) postoperative hospitalization with at least one overnight. For patients with multiple qualifying operations, the first surgery during the study period FY2006-FY2009 was used.

VA medical SAS datasets were used. These datasets are updated biweekly from nightly transmissions of patient utilization data system-wide (including the United States, its territories and possessions). Established protocols govern the formation of uniformly defined variables from each site. The validity of VA administrative databases has been ascertained by studies examining demographics, types of care, and diagnoses [18–20].

### Inpatient operations

CPT and ICD-9-A procedure codes identified operations. An iterative process conducted with physicians including surgeons during a 2-year pilot study determined 12,000 qualifying surgical procedures. Procedures conducted for diagnosis only were excluded as were non-surgical procedures, such as inpatient alcohol rehabilitation. Procedures were then grouped by type such as vascular operations.

### Serious mental illness

Serious mental illness was defined as in prior studies of VA patients. Diagnosis on two or more outpatient dates in the year prior to the qualifying index operation identified schizophrenia (ICD-9 codes 295.x excluding 295.5), bipolar disorder (296.0-296.1, 296.4-296.8), post-traumatic stress disorder (309.81), and major depressive disorder (296.2-296.3, 311) [21–23]. Patients treated for more than one serious mental illness were assigned the most frequently treated one, and in case of ties, the one highest in the psychotic hierarchy of schizophrenia, then bipolar disorder, then posttraumatic stress disorder, then major depression. For schizophrenia and bipolar disorder, one inpatient diagnosis was equally sufficient per the VA’s National Psychosis Registry [24]. Patients with other mental illness diagnoses in the range 290-311 were identified with an indicator (yes/no). Conditions specific to childhood (312-319) were not assessed being rare in veteran databases [25].

### Other measures

Covariates were chosen to adjust for baseline differences in physiologic reserve and socioeconomic status to the extent possible in the administrative medical data. Diagnosis codes from inpatient and outpatient records informed the summary measures of comorbidity over the one-year period before the date of the qualifying operation. The Charlson score of weighted indicators of 19 conditions associated with post-discharge one-year mortality [26, 27] and the Selim score of chronic physical conditions [28] as operationalized in VA administrative data by Pugh and colleagues [29] were both created. The Charlson contains 12 of the conditions captured by Selim physical, therefore they cannot be used in multivariable models together, however it is useful to assess the level of mortal conditions patients may have preoperatively. The Selim score of mental illness was not created as it contains most of the conditions VA categorizes as serious mental illness and therefore cannot be used in models containing the indicators for schizophrenia, bipolar disorder, PTSD, and major depression. Adverse outcomes included myocardial infarct, thromboembolytic events, pneumonia, respiratory failure, sepsis, wound infections and other postoperative complications per ICD-9 codes on inpatient records; readmission within 30 days of discharge; and death per the VHA Mini-Vitals file. This file of VHA patients’ dates of death has been validated against the National Death Index and demonstrated ~98 % sensitivity [30].

Demographics were collected from the administrative data extracts and aggregated to create indicators of Hispanic ethnicity, most commonly reported race, marital status and Census region in the year of index surgery. Previous studies have demonstrated imbalances in the distribution of surgeons, procedures, and postoperative mortality by geographic location hence we controlled for region [31–34]. Both race and ethnicity measures suffer from high levels of missing data in VA outpatient records. Age, gender and VA priority status were based on enrolment files. Because the VA preferentially enrols the most disadvantaged veterans and annually cares for about 5.5 million of the 22 million veterans living, a code for VA priority was established to determine eligibility to receive VA care and assessment of copays. Priority status is associated with both socioeconomic status and severity of illness acquired during military service [35]. In addition, because Priority one patients have no copays, priority measures one aspect of access to care [36]. Priority status includes (1) at least 50 % disabled by a military service-connected condition and no copays for care or drugs; (2-6) up to 40 % service-connected disability, special wartime cohorts such as those from Operations Enduring Freedom and Iraqi Freedom (OEF/OIF), catastrophically disabled, and impoverished, with pharmacy copays; and (7-8) other veterans agreeing to copays for both care and drugs.

### Analysis

The analyses examined the cohort of inpatient surgery patients and compared them to non-surgery patients using the VA during the same years, FY2006-FY2009. Descriptive and comparative statistics illustrated the types of procedures performed, the patients undergoing each type of operation, and postoperative readmission rates for patients with and without serious mental illness. Chi-square tests and analysis of variance assessed bivariate differences with a criterion alpha of .05. Logistic regression modelled relative odds of receipt of surgery in the total sample, as a function of SMI adjusting for clinical and demographic covariates. Covariates were gender, age, race including an indicator for missing data on race to retain full sample, region, priority 1 status, Selim chronic physical disease score, and indicators for nicotine dependence, obesity and dyslipidemia. Model fit was assessed by the c-statistic, which ranges from 0.5 (no better than chance) to 1.0 (perfect fit). Results were reported as odds ratio with 95 % confidence interval (OR [CI]). Odds ratios greater than 1.67 (i.e., a ratio of 4:3) or smaller than 0.75 (3:4) suggest a medium effect, and those greater than 2 (2:1) or smaller than 0.5 (1:2) denote a large effect [37]. The values between 0 and 1 (fractional values) represent “protective” effects or inverse associations with the outcome while values greater than one represent risk factors or positive associations with the outcome.

## Results

Over the 4-year period, the VA treated 7,150,127 veterans (priority 1-8), including 321,131 undergoing a major surgery qualifying for this study. The number of VA patients with serious mental illness totalled 907,088. The most common serious mental illness was PTSD, affecting 7 % of patients overall or having major surgery. The major surgery patients utilized a total of 3,814,994 inpatient days for the operations described; many went on to have additional admissions during the 4-year study period. More than half (56 %) of these inpatient days attributed to the index admission were consumed by the 7 % of the surgery sample (23,201 patients) with stays of 30 or more days (mean 92.8, SD 126.6). The average length of stay for the other 93 % of major surgery patients was 5.6 days (SD 5.6). Among the surgery patients, veterans of the Iraq and Afghanistan wars comprised 1 % (3016 persons); however, among the surgery patients with preoperative serious mental illness, the Iraq/Afghanistan veterans comprised 3 % (1342). Conversely, 31 % of Iraq/Afghanistan veterans undergoing surgery had preoperative serious mental illness compared to 14 % of surgery patients from other military cohorts.

### Differences by surgery vs non-surgery status

Surgery patients were more likely to be older, male, unmarried, African-American, impoverished, highly disabled, with a pre-existing psychotic disorder, and obese (all p < .05; see Table 1). Surgery patients had higher levels of chronic disease per Selim Physical but not of Charlson conditions. Eligibility for VA care was granted for poverty status to 39 % of surgery patients but 32 % of non-surgery patients. Being disabled 50-100 % (Priority 1) characterized 23 % of surgery patients and 12 % of non-surgery patients. These two priority categories described more than 60 % of surgery patients compared to 44 % of non-surgery patients. Non-surgery patients were more likely to have missing data on race (30 % vs 5 %) because inpatient data has more complete race data capture. The proportion of surgery patients with serious mental illness was 14 % vs 12 % non-surgery patients, with higher proportions of schizophrenia and bipolar disorder but comparable proportions of PTSD and MDD relative to non-surgery patients (Table 1).

**Table 1:** Characteristics of surgery and non-Surgery VA patients, fiscal years 2006-2009

Characteristic	Non-Surgery^a^ (*N* = 6,828,996)		Surgery (*N* = 321,131)	
	Mean	SD	Mean	SD
Age (range 18 -100 years)	60.8	17.0	63.7	12.2
	N	%	N	%
Women	376,738	5.5 %	15,620	4.9 %
Race †				
White	3,762,464	80.2 %	239,385	78.3 %
African American	810,744	17.3 %	58,617	19.2 %
Other	117,233	2.5 %	7834	2.6 %
Hispanic †	285,060	6.1 %	18,228	6.0 %
Married	3,450,809	55.7 %	151,250	47.1 %
Census Region ‡				
Northeast	933,339	15.0 %	39,381	12.3 %
Midwest	1,370,997	22.1 %	65,564	20.4 %
South	2,600,064	41.9 %	138,254	43.1 %
West	1,233,838	19.9 %	73,017	22.7 %
Puerto Rico and Virgin Islands	74,416	1.2 %	4915	1.5 %
VA Priority 1: 50-100 % service-connected disability	845,452	12.4 %	75,412	23.5 %
Obesity Diagnosis	329,231	5.3 %	89,624	27.9 %
Serious Mental Illness Group				
Schizophrenia	96,874	1.4 %	7155	2.2 %
Bipolar Disorder	102,801	1.5 %	6795	2.1 %
Post-Traumatic Stress Disorder	464,720	6.8 %	23,178	7.2 %
Major Depressive Disorder	172,441	2.5 %	8269	2.6 %
No Serious Mental Illness	5,992,160	87.8 %	275,734	85.9 %

^a^all comparisons significant at p < .0001

†31 % of non-surgery patients had missing data on race/ethnicity because of low rates of inpatient healthcare use; 8 % of major surgery patients had missing data on race/ethnicity

‡616,275 (9.1 %) non-surgery patients had missing data on Census Region

### Differences by serious mental illness status

Among the 321,131 surgery patients, 45,397 had been diagnosed with serious mental illness before their index operation. The overall rate of surgery among patients without mental illness was 4.5 %, significantly less than the 5.2 % surgery rate observed among patients with serious mental illness (p < .0001). There were significantly more women in the serious mental illness surgery groups (6 %-15 %) than in the non-mental illness surgery group (4 %) especially in the MDD and bipolar disorder groups (see Table 2). While women are more likely to have mental disorders such as bipolar disorder or depression [38], as veterans they are also more likely to come from more recent cohorts because of trends in military recruitment; they were therefore younger as a group. The surgery patients with serious mental illness were younger overall than the non-mental illness surgery patients: mean ages 56 to 60 years for our four serious mental illnesses vs. 65 years for other surgery patients (F = 2419.1; df = 4, 321126; p < .0001). Patients with serious mental illness were more likely to have a history of nicotine dependence (55-64 %) than other patients (46 %; p < .0001). Adverse outcomes varied somewhat by serious mental illness, with schizophrenia having the highest rates but no consistent pattern among the other groups (Table 2).

**Table 2:** Characteristics of VA surgery patients with or without serious mental illness, FY2006-FY2009 (*N* = 321,131)

	Schizophrenia		Bipolar Disorder		PTSD		MDD		Non-SMI	
	(*n* = 7155)		(*n* = 6795)		(*n* = 23,178)		(*n* = 8269)		(*n* = 275,734)	
Characteristic	N or mean	% or SD	N or mean	% or SD	N or mean	% or SD	N or mean	% or SD	N or mean	% or SD
Age^a^	59.9	10.7	56.1	10.9	59.2	10.1	58.6	11.2	64.6	12.2
Charlson Comorbidity Score^a^	2.6	2.6	2.1	2.5	2.4	2.5	2.5	2.6	2.8	2.7
Selim Chronic Disease Score^a^	6.2	2.5	6.3	2.5	6.5	2.4	6.1	2.4	4.7	2.4
Women^a^	429	6	994	14.6	1459	6.3	1042	12.6	11,696	4.2
Race										
Missing/unknown^a^	118	1.6	175	2.6	733	3.2	211	2.6	14,058	5.1
White^a^	4605	64.4	5509	81.1	17,047	73.5	6436	77.8	205,788	74.6
African American^a^	2241	31.3	928	13.7	4538	19.6	1369	16.6	49,541	18
Asian/Native American	191	2.7	183	2.7	860	3.7	253	3.1	6347	2.3
Hispanic^a^	655	9.3	285	4.3	1667	7.4	561	7	15,060	5.8
Married^a^	1576	22	2198	32.3	12,610	54.4	3311	40	131,555	47.7
Census Region										
Northeast^a^	1317	18.4	917	13.5	3101	13.4	1115	13.5	32,931	11.9
Midwest	1422	19.9	1551	22.8	3822	16.5	1886	22.8	56,883	20.6
South^a^	2554	35.7	2532	37.3	10,198	44	3275	39.6	119,695	43.4
West	1505	21	1752	25.8	5960	25.7	1797	21.7	62,003	22.5
Puerto Rico and Virgin Islands	357	5	43	0.6	97	0.4	196	2.4	4222	1.5
VA Priority 1 – 50-100 % service-connected disability^a^	3687	51.5	2305	33.9	16,428	70.9	2365	28.6	50,627	18.4
Obesity Diagnosis^a^	2357	32.9	2575	37.9	8739	37.7	3115	37.7	72,838	26.4
Complications of Surgery	655	9.2	531	7.8	1724	7.4	676	8.2	22,960	8.3
30-day Readmission	1129	15.8	899	13.2	2521	10.9	1048	12.7	32,521	11.8
30-day Postoperative Death	330	4.6	140	2.1	491	2.1	180	2.2	10,255	3.7

^a^all means for the serious mental illness groups were significantly different from those for no mental illness at p < .0001

Race/ethnicity differences were apparent but did not reflect a dichotomy with respect to serious mental illness; rather, patients with schizophrenia were more likely to be African-American or Hispanic compared to all other groups. Patients with conditions with psychotic features, i.e., schizophrenia or bipolar disorder, were less likely to be married than other patients. Regional differences were also evident, with higher concentrations of seriously mentally ill patients in the Northeast and West relative to other surgery patients. Finally, patients with serious mental illness had similar levels of diagnosed comorbidity with the exception of obesity which was relatively more common among mentally ill surgery patients.

### Types of operations

Over the 4-year study period, some of the most common types of surgery, accounting for at least 10 % of major operations, were digestive, vascular, hip-knee, lung-chest (non-cancer), and urogenital (see Table 3). Rates of CABG and vascular operations were lower among patients with serious mental illness especially those with schizophrenia. On the other hand, patients with schizophrenia were more likely to have operations to the skin and lungs/chest, and amputations such as partial foot removal.

**Table 3:** Relative rates of different types of inpatient operations for VA patients with vs without serious mental illness, FY2006-FY2009 (N = 321,131 Patients)

	All Surgery Patients		No Serious Mental Illness		Schizophrenia		Bipolar Disorder		Post-traumatic Stress Disorder		Major Depressive Disorder	
	(321,131)		(275,734)		(7155)		(6795)		(23,178)		(8269)	
Surgery Category	*N*	%	*N*	%	*N*	%	*N*	%	*N*	%	*N*	%
Vascular	38,786	12.1	34,876	12.6	506	7.1	469	6.9	2223	9.6	712	8.6
Coronary Artery Bypass Graft	18,269	5.7	16,265	5.9	237	3.3	270	4.0	1139	4.9	358	4.3
Cardiac	5927	1.8	5345	1.9	80	1.1	88	1.3	317	1.4	97	1.2
Hip/Knee	37,517	11.7	31,922	11.6	600	8.4	774	11.4	3221	13.9	1000	12.1
Musculoskeletal	23,139	7.2	23,139	8.4	546	7.6	660	9.7	2147	9.3	759	9.2
Back/Spinal Cord	20,480	6.4	20,480	7.4	343	4.8	627	9.2	2248	9.7	892	10.8
Integumentary System	18,806	5.9	15,931	5.8	681	9.5	484	7.1	1202	5.2	508	6.1
Hernia	14,005	4.4	11,939	4.3	347	4.8	348	5.1	987	4.3	384	4.6
Fractures	8019	2.5	6728	2.4	341	4.8	213	3.1	508	2.2	229	2.8
Amputation other than AKA/BKA	6464	2.0	5764	2.1	180	2.5	92	1.4	282	1.2	146	1.8
Below-Knee Amputation	1834	0.6	1636	0.6	46	0.6	20	0.3	93	0.4	39	0.5
Above-Knee Amputation	1489	0.5	1382	0.5	32	0.4	5	0.1	45	0.2	25	0.3
Digestive	40,248	12.5	34,866	12.6	1030	14.4	819	12.1	2596	11.2	937	11.3
Cholecystectomy	12,589	3.9	10,466	3.8	287	4.0	339	5.0	1060	4.6	437	5.3
Appendectomy	5920	1.8	4992	1.8	127	1.8	173	2.5	456	2.0	172	2.1
Lung/Chest	34,541	10.8	29,964	10.9	1098	15.3	721	10.6	2012	8.7	746	9.0
Nose/Mouth/Pharynx	5038	1.6	4187	1.5	95	1.3	119	1.8	467	2.0	170	2.1
Bone Marrow/Spleen/Liver	1875	0.6	1559	0.6	43	0.6	35	0.5	171	0.7	67	0.8
Transplant	632	0.2	574	0.2	2	0.0	10	0.1	36	0.2	10	0.1
Colorectal Cancer	16,381	5.1	14,621	5.3	339	4.7	262	3.9	861	3.7	298	3.6
Prostate Cancer	8906	2.8	7653	2.8	148	2.1	126	1.9	780	3.4	199	2.4
Lung Cancer	5183	1.6	4542	1.6	89	1.2	79	1.2	367	1.6	106	1.3
Head/Neck Cancer	3390	1.1	3024	1.1	54	0.8	53	0.8	193	0.8	66	0.8
Urinary/Male/Female Organs	30,362	9.5	25,927	9.4	615	8.6	736	10.8	2250	9.7	834	10.1
Lymph Nodes	13,534	4.2	11,856	4.3	251	3.5	212	3.1	941	4.1	274	3.3
Endocrine System	5475	1.7	4562	1.7	136	1.9	141	2.1	479	2.1	157	1.9

### Receipt of surgery

From the VHA system, 7,150,232 were analyzed. In the unadjusted model of receipt of major surgery as a function of serious mental illness, patients with schizophrenia (OR = 1.61 [CI 1.57-1.65]) or bipolar disorder (OR = 1.44 [1.40-1.47]) were more likely to have surgery; those with PTSD or major depression were marginally more likely to receive surgery (OR = 1.08 [1.07-1.10] for PTSD; OR = 1.04 [1.02-1.07] for depression). However, in adjusted models, patients with any serious mental illness were much less likely to receive major surgery, all other factors being equal (adjusting for demographic and clinical correlates, odds ratios ranged from 0.24-0.31 for the four disorders (95 % CI’s 0.24-0.25 to 0.30-0.31, p < .0001; Table 4). The fit was markedly better for the adjusted model (c-statistic = 0.87 on a scale of 0.50-1.00) than for the unadjusted model (c-statistic = 0.51). About 9 % of the sample had no records that could contribute diagnosis data; omitting these patients resulted in the same estimated effect sizes but a slightly poorer fit (c-statistic = 0.86; Table 4). In short, for patients with the same demographic-comorbidity profiles, having serious mental illness was associated with less major surgery.

**Table 4:** Parsimonious and full adjusted models of receipt of surgery among 7,150,126 patients in the Veterans health administration showing change in direction of effect of serious mental illness

Effect	Odds Ratio	95 % Confidence Limits	
Schizophrenia	1.61	1.57-1.65	
Bipolar disorder	1.44	1.40-1.47	
PTSD	1.08	1.07-1.10	
MDD	1.04	1.02-1.07	
c-statistic for unadjusted model: 0.51			
Schizophrenia	0.24	0.24-0.25	
Bipolar disorder	0.26	0.25-0.27	
PTSD	0.31	0.30-0.31	
MDD	0.29	0.28-0.30	
Female	1.16	1.14-1.18	
Age	0.99	0.99-0.99	
Black	0.97	0.96-0.98	
Other race (Asian/Native American)	0.86	0.84-0.89	
Missing data on race	0.45	0.45-0.46	
Hispanic	1.19	1.17-1.21	
VA priority 1 (no copays)	1.42	1.40-1.43	
Nicotine dependent	1.62	1.61-1.64	
Dyslipidemia	1.25	1.24-1.26	
Obesity diagnosis	1.00	0.98-1.01	
Selim physical comorbidity score	1.67	1.66-1.67	
c-statistic for adjusted model: 0.86			

### Adverse outcomes

Preliminary models of outcomes of surgery showed poor fit, with c-statistics ranging from 0.61 to 0.73 and modest associations with serious mental illness. Future work should seek to improve the models through restriction to specific types of surgery, such as cardiac operations,[39] and the inclusion of process of care variables such as lab testing and their results, tailored to specific types of operations.

## Discussion

The VA treated seven million veterans in FY2006-FY2009, including 4.5 % (321,131) who received inpatient surgical treatment; diagnostic operations and ambulatory procedures were excluded from this study. Among major surgery patients, 14 % met criteria for serious mental illness in the year prior to surgery, half of these for PTSD. In the VA, serious mental illnesses include PTSD, a diagnosis first appearing in the DMS-III in 1980. PTSD is both highly prevalent among post-combat veterans and deeply life-disturbing [40]. The frequency with which patients with serious psychiatric disorders undergo surgery appears to be largely undocumented [5]. Given the high prevalence of cardiovascular disease in patients with mental illness, strongly related to smoking and other lifestyle factors as well as some pharmaceutical side effects [41, 42], higher rates of surgical intervention among patients with serious mental illness might be expected. Yet in this comprehensive study of VA patients, operations to treat vascular disease and coronary artery blockage were relatively less common among patients with serious mental illness than among those without.

Patients with serious mental illness were much less likely to have major surgery, after controlling for age, other demographic measures, and disease burden. For patients of the same age, sex, race and comorbidity status, having a pre-existing serious mental illness conveyed a greatly decreased likelihood of surgical intervention. These patients may not be referred to surgical evaluation at the same rate, or may not present for timely referral and thus be deemed poor candidates for surgery.

Surgery patients were more likely to be highly disabled, impoverished, unmarried, and obese, relative to VA patients in general. Smoking, with its attendant cardiovascular risks, was noticeably more prevalent among surgery vs non-surgery patients although both groups showed unacceptably high rates of nicotine dependence, typical of post-military cohorts. This was seen in spite of known underreporting of tobacco histories from these administrative data which record only diagnoses and treatment rather than lifetime history [43]. Improved efforts at smoking cessation should be effected, regardless of mental disorders. Effective strategies in both smoking and dietary management have been demonstrated for patients with serious mental illness [44, 45].

To put the nature of the cohort in context, it is useful to realize that although veterans of US military service in general are healthier and wealthier than US residents overall, those veterans who use the VA are distinctly sicker and poorer [1]. Being disabled or obese is associated with poorer health status, while being married suggests both social support and potential health care coverage through a working spouse. This portrait of VA surgery patients reflects the department’s prioritization of providing care for those least likely to be able to receive care elsewhere.

Patients with serious mental illness were somewhat over-represented among surgery patients (14 %) relative to the VA patient population as a whole (12 %). These unadjusted results reflect what surgeons see in their operating rooms. The adjusted models that accounted for comorbidity reported under-representation of patients with serious mental illness among those getting surgery. This means that, for persons with equivalent levels of comorbidity, those with serious mental illnesses were less likely to get major surgical interventions. This may reflect clinician judgment that the patient would not manage postoperative care well, or patient eschewal of invasive definitive treatment, or low prioritization of addressing comorbid disease by patients and/or providers.

Veterans with high levels of service-connected disability and impoverished veterans were also relatively more likely to have major surgery. Disability, poverty, and serious mental illness often overlap: serious mental illness can be highly disabling and related to military service, and having serious mental illness greatly impedes a person’s ability to get or keep a job [46]. Taken together, these findings suggest that a lack of options coupled with reliance on the VA for care are likely the determining factors for where our most disadvantaged veterans undergo surgery. For the surgical team, including postoperative care team members, this suggests presence in the operating room of a sizable, high-risk subgroup requiring attentive follow-up to ensure good wound management and timely postoperative symptom reporting, activities some postoperative patients with serious mental illness will not be able to manage on their own. To reduce postoperative readmission with its attendant costs and increased risk of pain and death [47], innovative programs tailored to patients with serious mental illness may need to be developed and evaluated.

Post-traumatic stress disorder was strongly associated with high rates of procedures. Although veterans newly back from service in Operation Enduring Freedom (Afghanistan) or Operation Iraqi Freedom (the concluded combat actions in Iraq) are accessing the VA in large numbers, the majority of veterans being treated for PTSD are from the Vietnam Era [48], for the most part in their 60’s at the time of this study. Three percent of surgery patients were from the Iraq/Afghanistan conflicts, more than expected. The surfeit of operations and mental disorders among Iraq/Afghanistan veterans portends a long-term commitment of VA to these multiply challenged veterans, a commitment that will require careful planning to maintain VA’s high quality of care. These veterans of the Iraq/Afghanistan conflicts are the first to be admitted to VA without restriction other than deployment to hazardous arenas.

Schizophrenia in particular was associated with higher raw rates of postoperative 30-day mortality, in spite of these patients’ younger age. High comorbidity burden, disorganized and limited self-care capacity, potentially delayed presentation, and poor communication of symptoms are likely underlying factors for postoperative outcomes for these patients with psychotic illness. Our findings are thus not surprising but do suggest that follow-up care may need to be more aggressive and involve either outreach from VA or activation of caregivers for surgery patients with pre-existing serious mental illness. The data on patients with schizophrenia and bipolar disorder throughout our results fit with diminished ability to articulate symptoms (leading to lower treatment rates), while higher surgery rates associated with PTSD were consistent with comorbid pain and exposure to trauma [49].

The unhypothesized findings regarding region of the country suggested that VA patients in southern regions were more likely to have surgery, whether through choice of warmer climes during a period of declining health or endemic conditions increasing need for or provision of surgical intervention. Increased poverty and varying ethnic/racial distributions may also play a role in this result. Diagnosis with obesity was much more common in surgery patients, and obesity is known to be more common in the South [50] but this was not detectable in terms of diagnosed obesity in our surgery patients (results not shown). Understanding regional variation warrants careful examination in a future study.

Limitations of the study included reliance on VA data; data from out-of-system operations were not included. Results may not apply to persons with mental illness covered by private health insurance. The study relied on administrative data which do not designate operations as emergent, urgent, or elective. Serious mental illness was dependent on administrative coding which may misclassify some cases. It is possible that some underlying condition led to development of both prior-year serious mental illness and also the index surgery; lengthening the look-back period could address this issue but would restrict the sample with serious mental illness and reduce generalizability regarding proportions of VA patients at increased postoperative risk. In addition, the primary operation was not identified when a patient had multiple procedures on the same date.

## Conclusions

This is the first major study to look at system-wide rates of VA inpatient surgical treatment and its variation by serious mental illness. The findings supplement VASQIP data and enhance our understanding of not only surgery prevalence in the VA but factors associated with receipt of surgery among veterans. Further work of the STOPP project will examine other issues, including disparity by serious mental illness or other factors and outcomes of specific types of surgeries, and will delve into infrequently studied areas such as long-term outcomes of surgery. Policy implications include increased postoperative surveillance of patients with serious mental illness and perhaps greater availability and use of home health services or transitional care facilities such as long-term acute care.
